# Allergic Reactions to Brimonidine 0.15%: A Case Series

**DOI:** 10.7759/cureus.100095

**Published:** 2025-12-25

**Authors:** Fatin Nabila Mat Nawi, Mae-Lynn C Bastion

**Affiliations:** 1 Department of Ophthalmology, Faculty of Medicine, Universiti Kebangsaan Malaysia, Kuala Lumpur, MYS; 2 Department of Ophthalmology, Hospital Canselor Tuanku Muhriz, Kuala Lumpur, MYS

**Keywords:** allergic conjunctivitis, alphagan p, brimonidine 0.15%, glaucoma eyedrops, ocular allergy

## Abstract

Topical brimonidine is an effective anti-glaucoma agent and is usually well tolerated. This retrospective case series aimed to demonstrate the clinical features of brimonidine 0.15% (Alphagan P; Irvine, CA: Allergan Inc.) allergy. Five cases were found from one ophthalmologist’s two-year patient records. Patients’ ages ranged from 54 to 80 years. Three patients had ocular diagnoses of well-controlled primary open-angle glaucoma, one had normal-tension glaucoma, and another had ghost cell glaucoma. Brimonidine was combined with other anti-glaucoma medications for nine to 16 months before patients developed eye redness and swelling, which was not distressing. Itchiness was not a prominent symptom. Signs included follicles in the eyelid, bulbar conjunctiva with subtle chemosis, and a bumpy appearance. No patients exhibited marked changes in intraocular pressure during the allergy episode. None of the patients had self-discontinued brimonidine. In all cases, ocular symptoms improved dramatically upon withdrawal. One patient needed additional topical anti-histamine for relief. In brief, brimonidine allergy can occur months after commencement. Unlike other causes of allergic conjunctivitis, patients did not complain of itchiness, and bumpy conjunctival chemosis was a distinguishing feature. Ocular symptoms usually resolved upon cessation of brimonidine.

## Introduction

Brimonidine, an alpha-2 adrenergic agonist utilized as an anti-glaucoma ophthalmic solution, effectively reduces intraocular pressure (IOP) by 20-30% and is generally well-tolerated. Nonetheless, adverse effects, including ocular allergy, blurred vision, xerostomia, headache, drowsiness, and hypotension, may occur, potentially leading to medication discontinuation [1-3]. Alphagan P 0.15% was introduced as an alternative to Alphagan 0.2% to improve ocular tolerability by lowering the brimonidine concentration and replacing benzalkonium chloride with the Purite preservative; however, clinically significant ocular allergy continues to be reported.

Allergic follicular conjunctivitis represents the most common manifestation of ocular allergy, presenting with symptoms such as eye itchiness, foreign body sensation, lacrimation, and conjunctival hyperemia [3]. It was thought that adrenergic agonists reduce the volume of conjunctival cells, causing widening of intracellular spaces, thus allowing potential allergens to enter and result in a follicular reaction [4].

The reported incidence of brimonidine allergy varies from 4.7% to 25% in Western populations [5], with onset typically between six and nine months after initiation [2,6-8]. However, reported periods can range from 14 days to 12 months [9,10]. This delayed presentation, coupled with often subtle or nonspecific symptoms and the frequent presence of polypharmacy in glaucoma patients, may result in under-recognition of brimonidine allergy in clinical practice.

The purpose of this case series is to describe the clinical features of brimonidine 0.15% (Alphagan P; Irvine, CA: Allergan Inc.)-induced ocular allergy, with particular emphasis on delayed onset, subtle presenting signs, prolonged duration of use prior to diagnosis, and the lack of spontaneous medication discontinuation by patients.

## Case presentation

This retrospective case series involved a review of medical records from a single ophthalmologist’s clinic at the Universiti Kebangsaan Malaysia Specialist Clinic. The study focused on patients treated with brimonidine tartrate 0.15% preserved with Purite (Alphagan P; Irvine, CA: Allergan Inc.). Brimonidine allergy was defined by the presence of bulbar or tarsal conjunctival follicular reactions, which resolved upon cessation of brimonidine. Data recorded included patient demographics, type of glaucoma, concurrent glaucoma medication use, allergic symptoms, and duration of brimonidine use prior to allergy onset. Descriptive analysis was employed.

Five patients, ranging in age from 54 to 80 years (three males, two females), were identified. Diagnoses comprised three cases of well-controlled primary open-angle glaucoma (POAG), one normal-tension glaucoma (NTG), and one ghost cell glaucoma. Table 1 presents a summary of five cases.

**Table 1:** The clinical details of five cases. BE: both eyes; POAG: primary open-angle glaucoma; RE: right eye; LE: left eye; NTG: normal-tension glaucoma

Case	Age (years)/gender	Diagnosis	Medications	Symptoms	Duration on Alphagan P
1	80/female	BE POAG	G. Brimonidine tartrate P (Alphagan P) 0.15% BD BE, G. travaprost+timolol (Duotrav) ON BE, G. dorzolamide (Trusopt) BD BE	Eye redness, swelling, and itchiness	12 months
2	74/male	BE NTG	G. Brimonidine tartrate P 0.15% BD BE, G. bimatoprost (Lumigan) ON BE, G. dorzolamide (Trusopt) BD BE	Eye redness and itchiness	Many years
3	72/female	BE POAG	G. Brimonidine tartrate P 0.15% BD RE/TDS LE, G. brinzolamide (Azopt) BD RE/TDS LE, G. latanoprost (Xalatan) ON BE, G. timolol BD BE	Eye redness and discharge	12 months
4	68/male	BE POAG	G. Brimonidine tartrate P 0.15% BD BE, G. timolol BD BE	Eye itchiness and stinging	9 months
5	54/male	Ghost cell glaucoma	G. Brimonidine tartrate P 0.15% BD BE, G. dorzolamide (Trusopt) BD LE, G. timolol BD LE	Eye redness and itchiness	16 months

Patient one presented with a two-year history of bilateral progressive blurring of vision. Examination revealed right eye relative afferent pupillary defect with reduced best-corrected visual acuity (BCVA) (RE: 6/24, LE: 6/15), elevated intraocular pressures (RE: 33 mmHg, LE: 25 mmHg), and pale optic discs with RE cup disc ratio (CDR) of 0.9 and LE CDR of 0.6. Bilateral POAG was diagnosed, with advanced disease in the right eye, and treated initially with Duotrav for both eyes and Alphagan P for the right eye, achieving intraocular pressure (IOP) reduction below 15 mmHg. After 12 months, the patient developed persistent eye redness and itchiness with the presence of conjunctival follicles and papillae. She has a known allergy to seafood. She was given olopatadine for symptomatic relief. Significant improvement in ocular redness occurred within two months of discontinuing Alphagan P, supporting a diagnosis of drug-induced ocular reaction.

Patient two is an elderly diabetic male, not known to have any allergy before, with quiescent proliferative diabetic retinopathy (PDR), presented with visual acuity of 6/6 in the RE and 6/12 in the LE, IOP of 20 mmHg and 17 mmHg, respectively, and bilaterally pale optic discs. He was later initiated on bimatoprost (Lumigan) when repeat IOP measurements increased to 22 mmHg in the RE and 18 mmHg in the LE, while intermittently using Alphagan P on his own for several years. Six months later, he developed bilateral conjunctival injection with papillary reaction despite controlled IOP (16 mmHg in both eyes). The ocular allergy was initially unrecognized, and Alphagan P therapy was continued, delaying withdrawal for seven months. Resolution of ocular redness occurred within three weeks after discontinuation of Alphagan P.

Patient three, with no known allergy, was previously managed at another private center. At first presentation, the patient was on four anti-glaucoma agents, including Alphagan P, with a best-corrected visual acuity of 6/9.5 bilaterally, intraocular pressures of 12 mmHg in both eyes, and advanced optic disc cupping (CDR: 0.8 in the right eye and 0.9 in the left eye). After 12 months of follow-up, the patient developed marked conjunctival injection with papillary reaction. A regimen change to brinzolamide (Azopt) and tafluprost/timolol preservative-free combination (Tapcom S) resulted in complete resolution of allergy symptoms, with the patient reporting improved comfort and satisfaction.

Next is a POAG patient presented with ocular dryness while on bimatoprost/timolol (Ganfort). There was no documented ocular or systemic allergy history. Examination showed BCVA of 6/7.5 bilaterally, IOP of 15 mmHg in both eyes, optic disc with glaucomatous cupping (CDR: 0.7 right; 0.8 left), and signs of ocular surface toxicity, including lid hyperpigmentation, deepened eyelid sulcus, conjunctival injection, and superficial punctate keratopathy. Ganfort was discontinued and therapy was switched to topical timolol and Alphagan P twice daily. Nine months later, the patient developed eye itchiness and stinging symptoms, which resolved promptly following cessation of Alphagan P.

**Figure 1:**
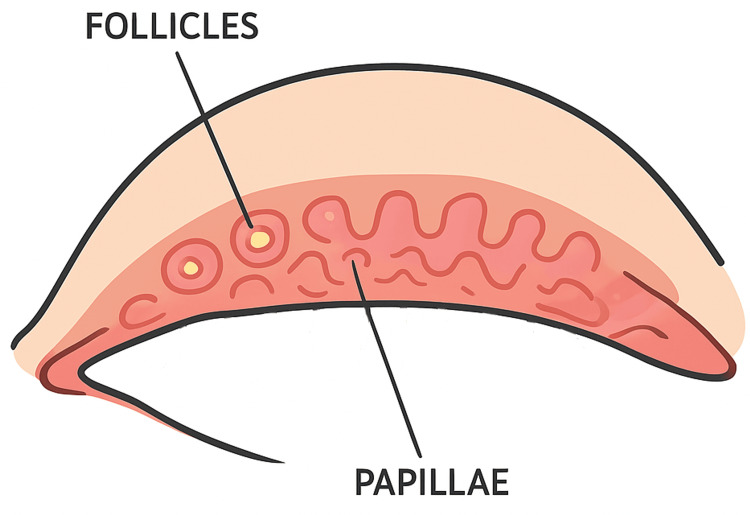
Schematic diagram showing follicles and papillae in upper eyelid (everted lid). This image was created by the main author of this study.

Last case is a diabetic and hypertensive patient with previously lasered proliferative diabetic retinopathy and bilateral cataracts, presented with reduced visual acuity (RE: 6/20, LE: 6/30) and IOP of 15 mmHg in both eyes. No prior history of ocular or systemic allergy was documented. The RE underwent cataract surgery when vision deteriorated to counting fingers, improving to 6/15 postoperatively. Subsequently, the LE developed hand movement vision, IOP of 50 mmHg, rubeosis iridis, and vitreous hemorrhage (VH), consistent with ghost cell glaucoma. Despite treatment with oral acetazolamide and four topical anti-glaucoma agents, including Alphagan P, IOP remained elevated at 36 mmHg, necessitating pars plana vitrectomy (PPV) and VH clearance. A similar course occurred in the RE four months later, requiring similar surgery. Three months after RE surgery and six months after LE, IOP stabilized at 15-16 mmHg, with pale optic discs and CDR 0.7 bilaterally. After 16 months of continued Alphagan P use, the patient developed bilateral conjunctival redness, itching, and papillary reaction, which resolved following drug discontinuation, indicating Alphagan P-induced ocular surface allergy. Figure 1 shows a schematic of eyelid follicles and papillae to help readers visualize the brimonidine ocular allergy observed in our case series.

In summary, all patients were concurrently using multiple anti-glaucoma agents. Four patients developed allergic symptoms after nine to 16 months of Alphagan P use, while one patient had been using the medication for several years. Predominant symptoms included ocular redness and eyelid swelling; ocular itching and discharge were minimal. Clinical signs observed were follicular reactions involving the eyelid and bulbar conjunctiva, accompanied by subtle conjunctival chemosis and a characteristic bumpy appearance. None of the patients discontinued brimonidine independently. Ocular symptoms, especially conjunctival redness, are markedly improved following withdrawal of brimonidine by treating ophthalmologist, with one patient receiving adjunctive topical anti-histamine therapy for symptom relief. 

## Discussion

Brimonidine reduces intraocular pressure by decreasing aqueous humor production and enhancing uveoscleral outflow. The original formulation, brimonidine tartrate 0.2% (Alphagan P; Irvine, CA: Allergan Inc.), contained benzalkonium chloride (BAK) as a preservative and was associated with side effects including xerostomia, conjunctival hyperemia, burning or stinging sensations, headache, blurred vision, and drowsiness in approximately 10-30% of patients, along with hypersensitivity reactions, such as allergic conjunctivitis and blepharoconjunctivitis in 5-14% of users [11]. Laboratory studies have demonstrated that BAK-preserved glaucoma medications exert dose-dependent toxic effects on conjunctival cells [12].

The medication was subsequently reformulated to brimonidine tartrate 0.15% (Alphagan P), containing Purite, a preservative with minimal toxicity that degrades into natural tear components [13,14]. Despite this change, allergic conjunctivitis, hyperemia, and ocular pruritus remain frequent adverse effects, occurring in approximately 10-20% of users, while burning sensation, oral dryness, and visual disturbance occur in about 5-9% of patients [11].

The pathophysiology of brimonidine-induced ocular allergy is not fully understood but is thought to involve immunologically mediated mechanisms. Adrenergic agonists may reduce conjunctival epithelial cell volume, widening intercellular spaces and facilitating allergen penetration, thereby triggering a follicular response [4]. Delayed-type hypersensitivity reactions have also been proposed, supported by the characteristic delayed onset and follicular conjunctivitis observed in many patients.

Findings from this series corroborate earlier reports indicating that allergic conjunctivitis and hyperemia are the most common adverse effects. Another study, however, reported no statistically significant differences between brimonidine Purite 0.15% and brimonidine 0.2% in the overall incidence of adverse events, and noted that conjunctival hyperemia and allergic conjunctivitis were the most frequently reported reactions, typically mild in severity [15]. Blondeau and Rousseau reported that 50% of patients who developed a brimonidine allergy had follicular conjunctivitis, a finding consistent with this case series [6].

Allergic reactions may also compromise IOP control, resulting in elevated values up to 28 mmHg or increases of approximately 1.25 mmHg, possibly due to increased conjunctival and episcleral blood flow associated with follicular conjunctivitis [5,10]. Transition to alternative anti-glaucoma agents is recommended to alleviate symptoms and maintain IOP control. Most patients in our study were on multiple topical medications, making IOP elevation after brimonidine allergy, if present, less obvious.

Most allergic reactions manifest within 12 months of brimonidine initiation [5,10], with peak incidence between six and nine months [2,6,7]. However, earlier onset as early as two weeks or later onset up to two years has been reported [5,16]. The duration of brimonidine use prior to allergy development does not differ between Alphagan BAK 0.2% and Alphagan Purite 0.15% [5]. In our cases, patients had been on Alphagan P for longer durations, making identification of the causative agent more challenging, particularly because they were concurrently using multiple anti-glaucoma medications. Concurrent use of timolol, a beta-adrenergic blocker, may reduce the incidence of brimonidine allergy by causing vasoconstriction and minimizing conjunctival hyperemia; three patients in this series were on timolol [17-19].

Conversely, prostaglandin analogs, used by three patients, may exacerbate allergic responses through proinflammatory activity and vasodilation, although their influence on brimonidine-related hypersensitivity has not been specifically investigated [20]. Clinical implications of this case series include the need for clinicians to maintain a high index of suspicion for brimonidine allergy in patients presenting with new-onset follicular conjunctivitis, conjunctival hyperemia, or ocular discomfort, particularly after prolonged exposure. Patients should be monitored for allergic reactions for at least the first 12 months of therapy, with awareness that delayed presentations may occur beyond this period. Prompt recognition, discontinuation of the suspected agent, and substitution with alternative glaucoma medications are essential for symptom resolution and preservation of intraocular pressure control.

This study has several limitations. The small sample size and retrospective design limit generalizability. Mild or nonspecific allergic symptoms may have been underreported, and no standardized grading system was used to assess allergy severity. The use of multiple concurrent glaucoma medications represents a potential confounding factor, complicating attribution of allergic reactions to brimonidine alone. In addition, objective confirmation of allergy, such as rechallenge or patch testing, was not performed in any of the cases.

## Conclusions

Brimonidine allergy may develop several months following commencement of Alphagan P therapy. Unlike other causes of allergic conjunctivitis, ocular pruritus may be absent. The presence of bumpy conjunctival chemosis serves as an important diagnostic indicator and needs to be evaluated in patients on long-term Alphagan P with unexplained redness. Ocular symptoms generally resolve upon discontinuation of brimonidine. Awareness and early recognition of this adverse reaction are essential to enable timely drug withdrawal, avoid unnecessary investigations or misattribution to other causes, and ensure patients’ comfort.
